# Identification of Bioactive Peptides from a *Laminaria digitata* Protein Hydrolysate Using In Silico and In Vitro Methods to Identify Angiotensin-1-Converting Enzyme (ACE-1) Inhibitory Peptides

**DOI:** 10.3390/md21020090

**Published:** 2023-01-27

**Authors:** Diane Purcell, Michael A. Packer, Maria Hayes

**Affiliations:** 1 Food BioSciences Department, Teagasc Food Research Centre, Ashtown, Dublin 15, D15 DY05 Dublin, Ireland; 2 Cawthron Institute, 98 Halifax Street, Nelson 7010, New Zealand

**Keywords:** *Laminaria digitata*, ACE-1 inhibition, bioactive peptides, protein hydrolysate, brown seaweed, in silico analysis

## Abstract

Bioactive peptides range in size from 2–30 amino acids and may be derived from any protein-containing biomass using hydrolysis, fermentation or high-pressure processing. Pro-peptides or cryptides result in shorter peptide sequences following digestion and may have enhanced bioactivity. Previously, we identified a protein hydrolysate generated from *Laminaria digitata* that inhibited ACE-1 in vitro and had an ACE-1 IC_50_ value of 590 µg/mL compared to an ACE-1 IC_50_ value of 500 µg/mL (~2.3 µM) observed for the anti-hypertensive drug Captopril©. A number of peptide sequences (130 in total) were identified using mass spectrometry from a 3 kDa permeate of this hydrolysate. Predicted bioactivities for these peptides were determined using an in silico strategy previously published by this group utilizing available databases including Expasy peptide cutter, BIOPEP and Peptide Ranker. Peptide sequences YIGNNPAKGGLF and IGNNPAKGGLF had Peptide Ranker scores of 0.81 and 0.80, respectively, and were chemically synthesized. Synthesized peptides were evaluated for ACE-1 inhibitory activity in vitro and were found to inhibit ACE-1 by 80 ± 8% and 91 ± 16%, respectively. The observed ACE-1 IC_50_ values for IGNNPAKGGLF and YIGNNPAKGGLF were determined as 174.4 µg/mL and 133.1 µg/mL. Both peptides produced sequences following simulated digestion with the potential to inhibit Dipeptidyl peptidase IV (DPP-IV).

## 1. Introduction

Bioactive peptides are sequences of amino acids ranging in size from 2–30 in length providing health benefits beyond basic nutrition when consumed [1]. Health benefits associated with bioactive peptides are extensive and include the reduction of hypertension and associated illnesses such as stroke and heart attack. The bioactive peptide activity pathway is thought to occur through the inhibition of enzymes within the Renin-Angiotensin-Aldosterone-System (RAAS) including Angiotensin-Converting-Enzyme-1 (ACE-1; EC3.4.15.1) and Renin (EC 3.4.23.15) [2,3,4,5]. In addition, other potential health benefits associated with bioactive peptides include anti-microbial and anti-inflammatory benefits, prevention of type 2 diabetes (T2D) through inhibition of alpha amylase (EC 3.2.1.1) and dipeptidyl peptidase IV (DPP-IV; EC 3.4.14.5) and inhibition of enzymes such as Prolyloligopeptidase (POP; EC 3.4.21.26) and BACE-1 that may result in mental health benefits. These peptides can be derived from any protein source including non-food sources such as natural protein produced in the gastrointestinal tract, but the most well-recognized sources are dairy, meat and fish [6,7].

Recently, we utilized enzymatic hydrolysis combining the enzymes Viscozyme^®^ and Alcalase^®^ as a method to extract protein from the brown seaweed *Laminaria digitata* [8]. Hydrolysis is a well-known strategy for the generation of bioactive peptides. Hydrolysis may increase the health benefits of protein hydrolysates. Several ACE-1 inhibitory peptides were identified to date from products including cheese, milk and yoghurt. ACE-1 is a zinc metallic protease, which converts Angiotensin I to the potent vasoconstrictor Angiotensin II, and enhances the degradation of the vasodilator Bradykinin [9]. Arterial Hypertension (AHT) is treated using drugs that inhibit the Angiotensin I-Converting Enzyme (ACE-1; EC 3.4.15.1) such as Enalapril^®^ or Captopril© [10,11,12,13]. However, side effects associated with these drugs have prompted research for natural remedies that may also treat or prevent the development of high blood pressure [14,15,16,17]. Previous studies have shown the ACE-1 and anti-hypertensive activities of the brown seaweeds *Undaria pinnatifida, Sargassum siliquosum and Sargassum polycystum*, [18,19].

In silico analysis is a valuable technique for predicting the potential bioactivities of peptides [3,20,21,22]. In silico analysis was used recently to identify anti-thrombotic [23,24,25] peptides from a variety of sources including dairy, mealworms, plants, peas, canola, maize and, more recently, seaweeds [20,26,27,28]. This approach has not, to the best of the authors’ knowledge, been applied to the brown seaweed *Laminaria digitata*.

This paper details the identification of peptides generated through hydrolysis of *Laminaria digitata* protein using enzymes and the characterisation of peptide sequences using mass spectrometry (MS). Subsequently, identified peptide sequences were ranked for potential bioactivities using an in silico approach described herein. One hundred and thirty peptides were identified from the *L. digitata permeates* and two peptides were synthesized. The ACE-1 IC_50_ values of these peptides were subsequently determined.

## 2. Results

### 2.1. Identification of Peptides Using Mass Spectrophometery and In Silico Analysis of Sequenced Peptides

A total of 130 peptides were identified from the 3-kDa permeate fraction using mass spectrometry (MS) as shown in Table 1 (*n* = 3). The identified peptide sequences had a >95% confidence level as being derived from the identified proteins listed in Table 2 and homology was confirmed using UniProt (https://www.uniprot.org/, accessed on 10 December 2022) [29]. Peptides had amino acid sequence homology with proteins from red seaweeds including *Neopyriopia yezoensis, Porphyra umbilicalis* and *Sporolithon durum*, and from the brown seaweeds including *Laminaria digitata*; *Colpomenia wynnei; Dictyopteris divaricate; Fucus vesiculosus, Ascophyllum nodosum; Sargassum horneri; Ectocarpus siliculosus; Carpomitra costata; Coccophora langsdorfii, Asterocladon rhodochortonoides; Choristocarpus tenellus; Asteronema ferruginea, Cladosiphon okamuranus and Tilopteris* mertensii as well as the red microalga *Porphyridium purpureum*, a marine bacterium *Tamlana fucoidanivorans* and the alpha proteobacteria *Pseudooceanicola algae.* The software programme Peptide Ranker (http://distilldeep.ucd.ie/PeptideRanker/, accessed on 10 December 2022) [30] identified peptides with potential bioactivities. Ten peptides including peptide IGNNPAKGGLF corresponding to amino acid peptide sequence f(315–326) of protein with accession number UniProtKB_Q1XDG4 (PBSS_NCOYE) derived from *Neopyriopia yezoensis* and peptide YIGNNPAKGGLF corresponding to f(314–326) of a protein with accession number UniProtKB_P51322 (PSBB_PORPU) from *Porphyra purpurea* were identified. Peptide DAALDFGPAL derived from the protein OX = 1537215 UniProt KB-A0A4185KT7_9RHOB and peptide AFYDYIGNNPAKGGLF from protein UniProtKB-Q1XDG4 (PSBB_NEOYE), and following peptides, SDGKIFDPL (UniProtKB_A0A6H5TY18 (A0A6H5J418_9PHAE); QGRVPGDIGFDPL (UniProtKB-A0A6HSJUW7_9PHAE); SMSGHPGAPM (UniProtKB_10A6H5L712_9PHAE); SEFIGFPIK (Uni-ProtKB-A0H6H5L026_9PHAE); and the final peptide GDFGNKDGKLTF (Uni-ProtKB-D8LG03) are all listed in Table 1, were identified. Identified peptides varied in length from 9–16 amino acids and had Peptide Ranker scores ranging from 0.64–0.82 (Table 1).

#### 2.1.1. Peptide Ranker

Peptide Ranker (http://distilldeep.ucd.ie/PeptideRanker/, accessed on 24 November 2022) is an open source software resource, which can be used to predict the potential bioactivity of peptides based on a novel N-to-1 neural network. Any user can submit peptides to Peptide Ranker, which will be returned to the user ranked by the probability that the peptide will be bioactive. It is important to note that this is not a prediction of the probability that the peptide has bioactivity [30].

Identified peptide IGNNPAKGGLF had a Peptide Ranker score of 0.82 which was the highest value obtained for any peptide identified using MS from the *L. digitata* 3 kDa permeate. This indicates that this peptide likely has bioactivity. Acceptable probability values for bioactivity are between 1.0–0.5. The peptide YIGNNPAKGGLF had a Peptide Ranker score of 0.81, indicating high potential bioactivity (Table 1). Peptides DAALDFGPAL and AFYDYIGNNPAKGGLF had Peptide ranker scores of 0.78. Peptide SDGKIFDPL had a score of 0.74 (Table 1).

#### 2.1.2. BIOPEP

A search of the BIOPEP database (https://biochemia.uwm.edu.pl/biopep-uwm/, accessed on 10 December 2022) [46] determined the novelty of the peptides identified and shown in Table 1. Of the ten peptides analyzed and listed in Table 2, their amino acid sequences were not identified in previously published papers concerning seaweed proteins and bioactive peptides [31,32,33,34,35,36,37,38,39,40,41,42,43,44,45].

#### 2.1.3. Simulated Digestion Using Peptide Cutter

Peptide cutter software (http://web.expasy.org/peptide_cutter/, accessed on 10 December 2022) [47] was used to determine if the identified peptides could potentially survive GI digestion. Peptides shown in Table 1 underwent simulated digestion using the GI tract enzymes, pepsin (pH 1.3), trypsin, and chymotrypsin. All peptides were cleaved into shorter peptide fragments that in some instances had known bioactivities and are found in BIOPEP (Table 2). Simulated GI digestion of the 10 peptides shown in Table 1 produced smaller peptides such as the active peptide GGL (derived following GI simulated digestion from YIGNNPAKGGLF). GGL is an active fragment, is a known anti-microbial peptide found in BIOPEP and it also has alpha-glucosidase inhibitory activities seen previously in Iberian dry-cured ham [31]. Peptides associated with other bioactivities include DPP-IV inhibition for monopeptides I, L; ACE-1 inhibition for dipeptides GD, TF, DP [35,36,43,44,45]. The monopeptide F, is a hydrophobic aromatic, amino acid, and it is thought, to enhance anti-oxidant activity [31]. Peptide IGNNPAKGGLF was digested into 3 peptides with sequences of IGNNPAK; GG and F. When comparing the first two peptides sequences listed in Table 2, the monopeptide Y, was one of two differing peptides. This monopeptide, Y, is hydrophobic, an aromatic amino acid, with anti-oxidant and anti-microbial bioactivity [31,32,33].

Several bioactive peptides also result from simulated GI digestion of the peptide DAALDFGPAL. Following simulated GI digestion the peptides DAA and GPAL result. DAA is a known antimicrobial peptide sequence found in the peptide tenecin 1, an insect defensin peptide [32]. The dipeptide DY results from simulated digestion of AFYDYIGNNPAKGGLF. This dipeptide is a known ACE-1 inhibitory peptide [34].

Simulated GI digestion of peptide SDGKIFDPL produces peptides SDGK and DP. The dipeptide DP identified previously from the dark muscle of tuna is a known ACE-1 inhibitor that also has anti-hypertensive activity shown in rat studies previously [36].

The peptide QGR occurs following simulated GI digestion of QGRVPGDIGFDPL. This tripeptide has known anti-microbial activity [37]. The peptide PL results following simulated GI digestion of YDYIGNNPAKGGLF. This tripeptide has known anti-microbial activity, and PL is also an ACE-1 inhibitor [37,38].

#### 2.1.4. Toxicity Assessment Using In Silico Analysis

All 130 peptides identified using MS were assessed for their potential to be toxic using ToxinPred (https://webs.iiitd.edu.in/raghava/toxinpred2/batch.html, accessed on 10 December 2022) [48]. Of the 130 peptides, tested results indicate that no peptides have potential toxicity.

#### 2.1.5. Peptide Synthesis and ACE-1 Inhibition

The peptides IGNNPAKGGLF and YIGNNPAKGGLF were synthesized and assessed in vitro for their ability to inhibit ACE-1. The peptide IGNNPAKGGLF was found to inhibit ACE-1 by 80% and YIGNNPAKGGLF inhibited ACE-1 by 91% when assayed at a concentration of 1 mg/mL compared to the control Captopril^®^ assayed at a concentration of 0.05 mg/mL. The ACE-1 IC_50_ values determined for both peptides were 174.4 µg/mL and 133.1 µg/mL for IGNNPAKGGLF and YIGNNPAKGGLF, respectively.

## 3. Discussion

Ten different peptide sequences were identified from the *L. digitata* protein 3 kDa permeate using MS. The ACE-1 inhibitory activity of two of these peptides was confirmed using chemical synthesis and assessment in vitro for ACE-1 inhibition. Additionally, other bioactivities were predicted using in silico methods. The MS-sequenced peptides ranged in length from 9–15 amino acids. All identified peptides were novel based on a search of the BIOPEP database and the literature. Peptide Ranker values were obtained for all peptides and the peptides likely to have bioactivities are shown in Table 1. These peptides had Peptide Ranker values greater than 0.5.

Two peptides, with Peptide Ranker scores of 0.82 and 0.81 were selected for chemical synthesis. ACE-1 inhibition values were determined in vitro for these peptides with amino acid sequences IGNNPAKGGLF and YIGNNPAKGGLF. ACE-1 and IC_50_ results for these synthesized peptides were obtained. Peptide IGNNPAKGGLF inhibited ACE-1 by 80% and YIHNNPAKGGLF inhibited ACE-1 by 91% when assayed at 1 mg/mL. The ACE-1 IC_50_ value for IGNNPAKGGLF was 174.4 µg/mL (0.161 µM) ACE-1. Peptide YIGNNPAKGGLF had an IC_50_ value of 133.1 µg/mL (0.11 µM) compared to Captopril© with a documented ACE-1 IC_50_ value of 500 µg/mL (2.3 µM) [8]. Previous studies on marine cryptides, used Captopril© as a positive control with IC_50_ values of (1.79–15.1 nM) for ACE-1, and another drug Losartan was used as a negative control for ACE-II inhibition, and had IC_50_ values of (17.13–146 μM) [49]. The IC_50_ for Captopril© varies depending on application and extraction methods used, with an IC_50_ of 7.09 nM from visible spectrophotometric (VSP) and for high-performance liquid chromatography (HPLC), and an IC_50_ of 4.94 nM [50]. Common hypertensive drugs, using the ACE-1 mechanism of control include Captopril©, Enalapril, Tekturna and Rasilez [51].

Peptides with ACE-1 IC_50_ values ranging from 2.42–20.63 µM [52] were identified from protein hydrolysates generated from *Laminaria japonica* previously. The IC_50_ values obtained for our synthesized peptides are greater than ACE-1 IC_50_ values reported previously for peptides such as HR, extracted from a bovine hydrolysate with an ACE-1 IC_50_ of 0.19 mM [51]. The ACE-1 inhibitory activity of the synthesized peptides is greater than the value reported for the *L. digitata* hydrolysate and shows the potential of these peptides for potential use in the treatment of hypertension.

Simulated GI digestion increased the potential bioactivities of identified peptides and several peptides with alpha-glucosidase and anti-microbial activities were found. Inhibition of alpha-glucosidase reduces carbohydrate digestion, consequently decreasing carbohydrate content in blood, which improves human health outcomes regarding type 2 diabetes [31,53]. The dipeptide sequence SE, cleaved from the novel peptide SEFIGFPIK (shown in Table 2), has potential stimulating vasoactive substance release bioactivity, discovered in peptides sourced from casein and soy protein previously [43,53]. The anti-inflammatory peptide sequence IGF also results from the GI digestion of SEFIGFPIK. This tripeptide is found in the pepsin hydrolysis of hempseed protein [42]. The peptide GNK that is cleaved from sequenced peptide GDFGNKDGKLTF is found in the Arietin peptide-A known as Fibrinogen interaction inhibitor. The dipeptide TF is also cleaved from the same sequenced peptide and is a known ACE-1 inhibitor [43,44,45].

This work identified two novel ACE-1 inhibitory peptides with pharmaceutically relevant ACE-1 IC_50_ values. In addition, the array of bioactive peptides that result following simulated GI digestion demonstrates the potential bioactivities still to be harnessed from brown seaweed proteins in *L. digitata.*

Additional bioactivities were also identified from cryptides identified following simulated gastrointestinal (GI) digestion. These bioactivities included Dipeptidyl peptidase IV (DPP-IV) inhibition potential for peptide sequences SDGK and alpha-glucosidase inhibition potential of peptides GGL and IGNNPAK. Future work will involve the synthesis of these peptides and determination of their in vitro inhibitory activities as well as the determination of their relevant IC_50_ values. Inhibitors of DPP-IV and alpha-glucosidase enzymes are the key targets for the pharmaceutical sector for development of drugs to prevent or to control type 2 diabetes [T2D].

## 4. Materials and Methods

### 4.1. Mass Spectrophotometry (MS) Characterisation of 3kDa Permeates

Protein extraction and peptide enrichment using molecular weight cut-off (MWCO) filtration was performed prior to MS characterisation in accordance with the method outlined in [8].

Peptide fractions were prepared for MS characterisation using the Phoenix peptide clean-up kit 4X, manufactured by Peromics and following the clean-up method supplied by the manufacturer. Peptides were identified using a mass spectrometer nanoESI qQTOF (6600 plus TripleTOF, AB SCIEX, Framingham, MA, U.S.A.) using liquid chromatography and tandem mass spectrometry (LC–MS/MS). A total of 1 μL of microalgal permeate was loaded onto a trap column (3 μ C18-CL 120 Ᾰ, 350 μM × 0.5 mm; Eksigent) and desalted with 0.1% TFA (trifluoroacetic acid) at 5 μL/min for 5 min. The peptides were then loaded onto an analytical column (3 μ C18-CL 120 Ᾰ, 0.075 × 150 mm; Eksigent) equilibrated in 5% acetonitrile 0.1% FA (formic acid). Elution was carried out with a linear gradient from 7 to 45% B in A for 20 min, where solvent A was 0.1% FA and solvent B was ACN (acetonitrile) with 0.1% FA at a flow rate of 300 nL/min. The sample was ionized in an electrospray source Optiflow < 1 μL Nano applying 3.0 kV to the spray emitter at 200 °C. Analysis was carried out in a data-dependent mode. Survey MS1 scans were acquired from 350 to 1400 *m*/*z* for 250 ms. The quadrupole resolution was set to ‘LOW’ for MS2 experiments, which were acquired from 100 to 1500 *m*/*z* for 25 ms in ‘high sensitivity’ mode. Up to 50 ions were selected for fragmentation after each survey 400 scan. Dynamic exclusion was set to 15 s. The system sensitivity was controlled by analyz-401 ing 500 ng of K562 protein extract digest (SCIEX); in these conditions, 2260 proteins were identified (FDR < 1%) in a 45 min gradient. Peptides identified as having potential bioactivities were chemically synthesised by GenScript Biotech (Leiden, The Netherlands). GenScript also verified the purity of the peptides by analytical RP-HPLC–MS.

### 4.2. In Silico Analysis of MS Sequenced Peptides

Peptide Ranker was used to predict the bioactivity of peptide sequences and values of between 0.5 and 1 were taken as indicative of peptides having bioactivity.

**Figure 1:**
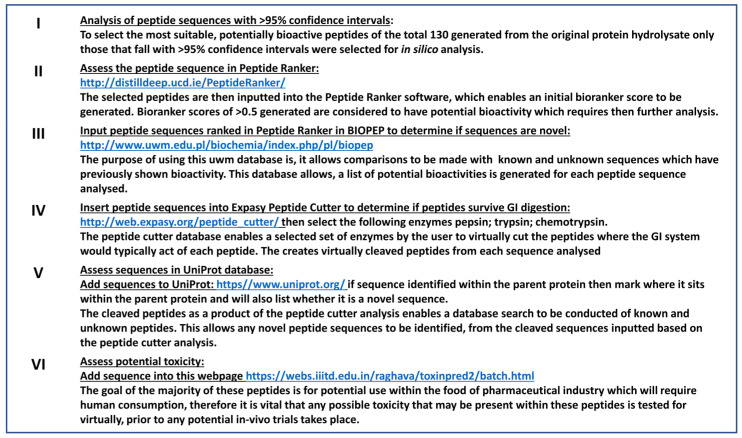
In silico methodology based on the method by Lafarga et al. 2014, Hayes et al., 2018, and Hayes et al., 2021 [3,6,7] was used for the identification and generation of ACE-I inhibitory peptides from *L. digitata* proteins. Information including the structure, amino acid sequence and composition of the proteins was collected. Peptide Ranker; BIOPEP; Expasy PeptideCutter Tool; UniProt and ToxinPred were used on the peptide sequences. Peptide Ranker and BIOPEP ranked the potentially most bioactive sequences and identified the bioactivities of these peptides. Expasy PeptideCutter Tool was used to predict the probable cleavage sites of selected enzymes within the top ten sequences listed in Table 1. ToxinPred was used for predicting the toxicity of peptides identified in this project.

Figure 1 shows the six steps used during in silico analysis. Of the 130 peptides identified using MS, only those with >95% confidence were selected for synthesis and in silico analysis. Selected peptides were input into the software programme Peptide Ranker (http://distilldeep.ucd.ie/PeptideRanker/, accessed on 15 December 2022). A value indicative of potential bioactivity was obtained for each peptide. Only peptides with Peptide Ranker scores greater than 0.5 were used in further analysis. Ten peptide sequences were identified as having potential bioactivities. The novelty of these peptides was determined following a search in the peptide database BIOPEP (http://www.uwm.edu.pl/biochemia/index.php/pl/biopep, accessed on 12 December 2022). Active peptides were further assessed for their ability to survive simulated GI digestion using Expasy peptide cutter (http://web.expasy.org/peptide_cutter/, accessed on 10 December 2022). The UniProt database was used to identify proteins containing the peptide sequences. Additionally, the potential toxicity of identified peptides was assessed using the software programme ToxinPred (https://webs.iiitd.edu.in/raghava/toxinpred2/batch.html, accessed on 10 December 2022).

### 4.3. ACE-1 Inhibitory Activity Assessment

The peptides with the highest Peptide Ranker scores IGNNPAKGGLF, with a peptide ranker value of 0.82, and YIGNNPAKGGLF, with a value of 0.81,were selected for synthesis. Once made, peptides were re-tested using in vitro screening assays. ACE-1 activity was tested using an assay kit supplied by Cambridge BioSciences (Cambridge, UK) as described previously. Captopril© (a known ACE-1 inhibitor) dissolved in distilled water was used as a positive control.

## 5. Conclusions

In silico and in vitro methods are useful tools for selection of enzymes to generate bioactive peptides from protein containing biomass. Moreover, they are useful to determine potential bioactivities of peptides prior to chemical synthesis and can save time and money prior to animal studies to determine potential health benefits. A combination of these methods was used previously to identify and confirm the bioactivity of peptides derived from blood proteins [51] and microalgae previously [54]. However, limitations of this approach exist and specifically include limits concerning the folding of protein, which has an impact on how enzymes cut the protein and which in turn can impact production of the resulting peptides. One of the main barriers for entering the human functional foods market is unknown and unstable peptide product qualities. It is required to have analytical methods for characterising the peptide fraction. Today, research groups are using Fourier-transform Infrared (FTIR) fingerprints to gain new insight in quality variations of peptide products. These fingerprints can be related to raw material composition and processing factors [55]. The method used in this study has advantages over in vitro only methods as it can help to predict the best enzymes to use to generate bioactive peptide containing hydrolysates and additionally can predict the most bioactive peptides and those that may be toxic before any in vitro assays are performed.

Two novel ACE-1 inhibitory peptides with amino acid sequences corresponding to IGNNPAKGGLF and YIGNNPAKGGLF were identified from a 3 kDa permeate of a protein hydrolysate generated from the brown seaweed *L. digitata.* In silico methods also predicted the potential of this seaweed as a source of novel, bioactive peptides that may impart additional health benefits to the consumer including prevention of T2D and antimicrobial activities following GI digestion. Identified, chemically synthesized peptides had ACE-1 inhibition IC_50_ values of 174.4 µg/mL (0.161 µM) for peptide IGNNPAKGGLF and 133.1 µg/mL (0.107 µM) for peptide YIGNNPAKGGLF and both peptides were similar in terms of bioactivity to other ACE-1 inhibitory peptides identified from tuna and meat muscle previously. This study highlights the potential bioactivity of this brown seaweed. However, future work is required to confirm an anti-hypertensive effect of the seaweed hydrolysate and synthesized peptides in vivo. This work will involve assessment of the *L. digitata* hydrolysate and synthesized peptides in spontaneously hypertensive rats (SHRs) to assess if the ACE-1 inhibitory peptides have an anti-hypertensive effect in vivo.

## Figures and Tables

**Table 1:** Identified peptide sequences from a *Laminaria digitata* protein hydrolysate 3 kDa permeate fraction. These were identified using MS, and the Peptide Ranker score indicates potential bioactivity (a score closer to 1 is indicative of bioactivity).

Cleaved Peptide Sequence	Peptide Ranker Value (Accessed on 10 December 2022), (#)
IGNNPAKGGLF	0.82
YIGNNPAKGGLF	0.81
DAALDFGPAL	0.78
AFYDYIGNNPAKGGLF	0.78
SDGKIFDPL	0.74
YDYIGNNPAKGGLF	0.73
QGRVPGDIGFDPL	0.67
SMSGHPGAPM	0.65
SEFIGFPIK	0.64
GDFGNKDGKLTF	0.64

# universal mathematical symbol for a number.

**Table 2:** Identified peptide sequences and potential bioactivities associated with a *Laminaria digitata* protein hydrolysate 3 kDa permeate fraction identified using MS and in silico analysis.

Parent Protein Name and UniProt Accession Number	Peptide Single Amino Acid Sequence	f(X–X) of Parent Protein	Peptide Ranker Value ^1^	Novelty (Found in BIOPEP ^2^ Database)	Observed Bioactivity	Simulated Digestion Using PeptideCutter ^3^/Peptide Digestion Fragments	Associated Predicted Bioactivities	References
*Neopyriopia yezoensis* NCBI Taxonomy ID 2788 Photosystem II CP47 reaction centre protein UniProtKB_Q1XDG4 (PBSS_NCOYE)	IGNNPAKGGLF	f(315–326)	0.82	Novel	ACE-1 inhibition	IGNNPAK; GGL; F	(GGL) Antimicrobial activity; Alpha-glucosidase inhibition	[31]
*Porphyra purpurea* NCBI Taxonomy ID P51322 Photosystem II CP47 reaction centre protein UniProtKB_P51322 (PSBB_PORPU)	YIGNNPAKGGLF	f(314–326)	0.81	Novel	ACE-1 inhibition	Y; IGNNPAK; GGL; F	Antimicrobial; Alpha-glucosidase inhibition	[31]
*Pseudooceanicola algae* OX = 1537215 UniProt KB-A0A4185KT7_9RHOB	DAALDFGPAL	f(53–62)	0.78	Novel	ACE-1 inhibition	DAA; L; D; F; GPAL	Antimicrobial	[32]
*Neopyropia yezoensis* (Susabi-nori) (*Pyropia yezoensis*) UniProtKB-Q1XDG4 (PSBB_NEOYE) Photosystem II CP47 reaction centre protein	AFYDYIGNNPAKGGLF	f(310–325)	0.78	Novel	ACE-1 inhibition	A; F; Y; DY; IGNNPAK; GGL; F	Antimicrobial; Alpha-glucosidase inhibition; ACE inhibitor	[33,34]
*Ectocarpus* species CCAP UniProtKB_A0A6H5TY18 (A0A6H5J418_9PHAE) LHCP protein	SDGKIFDPL	f(57–65)	0.74	Novel	ACE-1 inhibition	SDGK; I; F; DP; L	Dipeptidyl peptidase IV inhibitor; ACE inhibitor	[35,36]
*Neopyropia yezoensis* Photosystem II CP47 reaction centre protein PSbB UniProtKB_Q1XDG4	YDYIGNNPAKGGLF	f(312–325)	0.73	Novel	ACE-1 inhibition	Y; DY; IGNNPAK; GGL; F	Alpha glucosidase inhibition; ACE inhibitor; Antimicrobial	[31,34]
*Ectocarpus* sp. CCAP 1310/34 Uncharacterised protein UniProtKB-A0A6HSJUW7_9PHAE	QGRVPGDIGFDPL	f(151–163)	0.67	Novel	ACE-1 inhibition	QGR; VPGDIG; F; D; PL	Antimicrobial (QGR); ACE inhibitor; (PL) ACE inhibitor	[37,38,39]
*Ectocarpus* sp. CCAP 131-/34 Transketolase_1_domain containing protein UniProtKB_10A6H5L712_9PHAE	SMSGHPGAPM	f(37–46)	0.65	Novel	ACE-1 inhibition	SM; SGHPGAPM	DPP-III inhibitor (SM)	[40]
*Ectocarpus* sp. CCAP 131/34 HSP90 protein UniProtKB-A0H6H5L026_9PHAE	SEFIGFPIK	f(208–216)	0.64	Novel	ACE-1 inhibition	SE; F; IGF; PIK	Anti-inflammatory (IGF); Antimicrobial (PIK); Stimulating vasoactive substance release (SE)	[41,42]
*Ectocarpus siliculosus* (Brown algae) Manganese stabilising protein UniProtKB-D8LG03	GDFGNKDGKLTF	f(161–172)	0.64	Novel	ACE-1 inhibition	GD; F; GNK; DGK; L; TF	ACE inhibitor (GD); Fibrinogen interaction inhibitor (GNK-part of a peptide called Arietin); ACE inhibitor (TF)	[43,44,45]

^1^http://distilldeep.ucd.ie/PeptideRanker/, accessed on 10 December 2022, ^2^https://biochemia.uwm.edu.pl/biopep-uwm/, accessed on 10 December 2022, ^3^ software http://web.expasy.org/peptide_cutter/, accessed on 10 December 2022.

## Data Availability

Data are available from the corresponding author.

## References

[1] Ondetti M.F., Cushman D.W. (1982). Enzymes of the renin-angiotensin system and their inhibitors. Annu. Rev. Biochem..

[2] He Z., Liu G., Qiao Z., Cao Y., Song M. (2021). Novel Angiotensin-I Converting Enzyme Inhibitory Peptides Isolated from Rice Wine Lees: Purification, Characterization, and Structure-Activity Relationship. Front. Nutr..

[3] Lafarga T., O’Connor P., Hayes M. (2014). Identification of novel dipeptidyl peptidase-IV and angiotensin-I-converting enzyme inhibitory peptides from meat proteins using in silico analysis. Peptides.

[4] Goossens G.H. (2012). The Renin-Angiotensin System in the Pathophysiology of Type 2 Diabetes. Obes. Facts.

[5] Wang Y., Tikellis C., Thomas M.C., Golledge J. (2013). Angiotensin converting enzyme 2 and atherosclerosis. Atherosclerosis.

[6] Hayes M. (2018). Food Proteins and Bioactive Peptides: New and Novel Sources, Characterisation Strategies and Applications. Foods.

[7] Hayes M. (2021). Bioactive Peptides in Preventative Healthcare: An Overview of Bioactivities and Suggested Methods to Assess Potential Applications. Curr. Pharm. Des..

[8] Purcell D., Packer M.A., Hayes M. (2022). Angiotensin-I-Converting Enzyme Inhibitory Activity of Protein Hydrolysates Generated from the Macroalga Laminaria digitata (Hudson) JV Lamouroux 1813. Foods.

[9] Soffer R.L. (1976). Angiotensin-Converting Enzyme and the Regulation of Vasoactive Peptides. Annu. Rev. Biochem..

[10] Julius S., Nesbitt S.D., Egan B.M., Weber M.A., Michelson E.L., Kaciroti N., Black H.R., Grimm R.H., Messerli F.H., Oparil S. (2006). Feasibility of treating prehypertension with an angiotensin-receptor blocker. N. Engl. J. Med..

[11] Bhuyan B.J., Mugesh G. (2011). Synthesis, characterization and antioxidant activity of angiotensin converting enzyme inhibitors. Org. Biomol. Chem..

[12] Osterziel K.J., Dietz R., Harder K., Kübler W. (1992). Comparison of captopril with enalapril in the treatment of heart failure: Influence on hemodynamics and measures of renal function. Cardiovasc. Drugs Ther..

[13] Alan S.L.A., Yu M.B., Chir B., Chertow G., Luyckx V., Marsden P., Skorecki K., Maarten M., Yu A. (2020). Renovascular Hypertension and Ischemic Nephropathy. Brenner & Rector’s the Kidney.

[14] Lordan S., Ross R.P., Stanton C. (2011). Marine bioactives as functional food ingredients: Potential to reduce the incidence of chronic diseases. Mar. Drugs.

[15] Wijesekara I., Kim S.-K. (2010). Angiotensin-I-converting enzyme (ACE) inhibitors from marine resources: Prospects in the pharmaceutical industry. Mar. Drugs.

[16] Seca A.M.L., Pinto D.C.G.A. (2018). Overview on the Antihypertensive and Anti-Obesity Effects of Secondary Metabolites from Seaweeds. Mar. Drugs.

[17] Pujiastuti D.Y., Ghoyatul Amin M.N., Alamsjah M.A., Hsu J.-L. (2019). Marine Organisms as Potential Sources of Bioactive Peptides that Inhibit the Activity of Angiotensin I-Converting Enzyme: A Review. Molecules.

[18] Nagappan H., Pee P.P., Kee S.H.Y., Ow J.T., Yan S.W., Chew L.Y., Kong K.W. (2017). Malaysian brown seaweeds Sargassum siliquosumnand Sargassum polycystum: Low density lipoprotein (LDL) oxidation, angiotensin converting enzyme (ACE)—Amylase, and-glucosidase inhibition activities. Food Res. Int..

[19] Hata Y., Nakajima K., Uchida J.-I., Hidaka H., Nakano T. (2001). Clinical Effects of Brown Seaweed, *Undaria pinnatifida* (wakame) on Blood Pressure in Hypertensive Subjects. J. Clin. Biochem. Nutr..

[20] Vermeirssen V., van der Bent A., Van Camp J., van Amerongen A., Verstraete W. (2004). A quantitative in silico analysis calculates the angiotensin I converting enzyme (ACE) inhibitory activity in pea and whey protein digests. Biochimie.

[21] Udenigwe C.C., Gong M., Wu S. (2013). In silico analysis of the large and small subunits of cereal RuBisCO as precursors of cryptic bioactive peptides. Process Biochem..

[22] Hashemi Z.S., Zarei M., Fath M.K., Ganji M., Farahani M.S., Afsharnouri F., Pourzardosht N., Khalesi B., Jahangiri A., Rahbar M.R. (2021). In silico Approaches for the Design and Optimization of Interfering Peptides Against Protein–Protein Interactions. Front. Mol. Biosci..

[23] Chen F., Jiang H., Lu Y., Chen W., Huang G. (2019). Identification and in silico analysis of anti-thrombotic peptides from the enzymatic hydrolysates of Tenebrio molitor larvae. Eur. Food Res. Technol..

[24] Zengin G., Stefanucci A., Rodrigues M.J., Mollica A., Custodio L., Aumeeruddy M.Z., Mahomoodally M.F. (2019). Scrophularia lucida L. as a valuable source of bioactive compounds for pharmaceutical applications: In vitro anti-oxidant, anti-inflammatory, enzyme inhibitory properties, in silico studies, and HPLC profiles. J. Pharm. Biomed. Anal..

[25] Hayes M., Stanton C., Slattery H., O’Sullivan O., Hill C., Fitzgerald G.F., Ross R.P. (2007). Casein fermentate of Lactobacillus animalis DPC6134 contains a range of novel propeptide angiotensin-converting enzyme inhibitors. Appl. Environ. Microbiol..

[26] Cian R.E., Nardo A.E., Garzón A.G., Añon M.C., Drago S.R. (2022). Identification and in silico study of a novel dipeptidyl peptidase IV inhibitory peptide derived from green seaweed Ulva spp. hydrolysates. LWT.

[27] Díaz-Gómez J.L., Neundorf I., López-Castillo L.-M., Castorena-Torres F., Serna-Saldívar S.O., García-Lara S. (2020). In Silico Analysis and In Vitro Characterization of the Bioactive Profile of Three Novel Peptides Identified from 19 kDa α-Zein Sequences of Maize. Molecules.

[28] Duan X., Zhang M., Chen F. (2021). Prediction and analysis of anti-microbial peptides from rapeseed protein using in silico approach. J. Food Biochem..

[29] Consortium T.U. (2020). UniProt: The universal protein knowledgebase in 2021. Nucleic Acids Res..

[30] Mooney C., Haslam N.J., Pollastri G., Shields D.C. (2012). Towards the Improved Discovery and Design of Functional Peptides: Common Features of Diverse Classes Permit Generalized Prediction of Bioactivity. PLoS ONE.

[31] Mora L., González-Rogel D., Heres A., Toldrá F. (2020). Iberian dry-cured ham as a potential source of α-glucosidase-inhibitory peptides. J. Funct. Foods.

[32] Ren J., Zhao M., Shi J., Wang J., Jiang Y., Cui C., Kakuda Y., Xue S.J. (2008). Purification and identification of anti-oxidant peptides from grass carp muscle hydrolysates by consecutive chromatography and electrospray ionization-mass spectrometry. Food Chem..

[33] Rajapakse N., Mendis E., Byun H.-G., Kim S.-K. (2005). Purification and in vitro anti-oxidative effects of giant squid muscle peptides on free radical-mediated oxidative systems. J. Nutr. Biochem..

[34] Ziganshin R.H., Svieryaev V.I., Vas’kovskiĭ B.V., Mikhaleva I.I., Ivanov V.T., Kokoz Y.M., Alekseev A.E., Korystova A.F., Sukhova G.S., Emel’ianova T.G. (1994). Biologically active peptides isolated from the brain of hibernating ground squirrels. Bioorg. Khim..

[35] Wu J., Aluko R.E., Nakai S. (2006). Structural requirements of Angiotensin I-converting enzyme inhibitory peptides: Quantitative structure-activity relationship study of di- and tripeptides. J. Agric. Food Chem..

[36] Lan V.T., Ito K., Ohno M., Motoyama T., Ito S., Kawarasaki Y. (2015). Analyzing a dipeptide library to identify human dipeptidyl peptidase IV inhibitor. Food Chem..

[37] Qian Z.J., Je J.Y., Kim S.K. (2007). Anti-hypertensive effect of angiotensin i converting enzyme-inhibitory peptide from hydrolysates of Bigeye tuna dark muscle, *Thunnus obesus*. J Agric. Food Chem..

[38] Byun H.G., Kim S.K. (2002). Structure and activity of angiotensin I converting enzyme inhibitory peptides derived from Alaskan pollack skin. J. Biochem. Mol. Biol..

[39] Nogata Y., Nagamine T., Yanaka M., Ohta H. (2009). Angiotensin I Converting Enzyme Inhibitory Peptides Produced by Autolysis Reactions from Wheat Bran. J. Agric. Food Chem..

[40] Forghani B., Zarei M., Ebrahimpour A., Philip R., Bakar J., Abdul Hamid A., Saari N. (2016). Purification and characterization of angiotensin converting enzyme-inhibitory peptides derived from Stichopus horrens: Stability study against the ACE and inhibition kinetics. J. Funct. Foods.

[41] Dhanda S., Singh J., Singh H. (2008). Hydrolysis of various bioactive peptides by goat brain dipeptidylpeptidase-III homologue. Cell Biochem. Funct..

[42] Cruz-Chamorro I., Santos-Sánchez G., Bollati C., Bartolomei M., Li J., Arnoldi A., Lammi C. (2022). Hempseed (*Cannabis sativa*) Peptides WVSPLAGRT and IGFLIIWV Exert Anti-inflammatory Activity in the LPS-Stimulated Human Hepatic Cell Line. J. Agric. Food Chem..

[43] Ringseis R., Matthes B., Lehmann V., Becker K., Schöps R., Ulbrich-Hofmann R., Eder K. (2005). Peptides and hydrolysates from casein and soy protein modulate the release of vasoactive substances from human aortic endothelial cells. Biochim. Biophys. Acta.

[44] Cheung H.-S., Wang F.-L., Ondetti M.A., Sabo E.F., Cushman D.W. (1980). Binding of peptide substrates and inhibitors of angiotensin-converting enzyme. Importance of the COOH-terminal dipeptide sequence. J. Biol. Chem..

[45] Huang T.F., Holt J.C., Lukasiewicz H., Niewiarowski S. (1987). A low molecular weight peptide inhibiting fibrinogen interaction with platelet receptors expressed on glycoprotein IIb-IIIa complex. J. Biol. Chem..

[46] Minkiewicz P., Iwaniak A., Darewicz M. (2019). BIOPEP-UWM Database of Bioactive Peptides: Current Opportunities. Int. J. Mol. Sci..

[47] Gasteiger E., Hoogland C., Gattiker A., Duvaud S.E., Wilkins M.R., Appel R.D., Bairoch A., Walker J.M. (2005). Protein Identification and Analysis Tools on the ExPASy Server. The Proteomics Protocols Handbook.

[48] Gupta S., Kapoor P., Chaudhary K., Gautam A., Kumar R., Open Source Drug Discovery C., Raghava G.P.S. (2013). In Silico Ap-proach for Predicting Toxicity of Peptides and Proteins. PLoS ONE.

[49] Henda Y.B., Labidi A., Arnaudin I., Bridiau N., Delatouche R., Maugard T., Piot J.-M., Sannier F., Thiéry V., Bordenave-Juchereau S. (2013). Measuring Angiotensin-I Converting Enzyme Inhibitory Activity by Micro Plate Assays: Comparison Using Marine Cryptides and Tentative Threshold Determinations with Captopril and Losartan. J. Agr. Food Chem..

[50] Chen J., Wang Y.R., Wu Y., Xia W. (2013). Comparison of analytical methods to assay inhibitors of angiotensin I-converting enzyme. Food Chem..

[51] Lafarga T., Rai D.K., O’Connor P., Hayes M. (2016). Generation of Bioactive Hydrolysates and Peptides from Bovine Hemoglobin with In Vitro Renin, Angiotensin-I-Converting Enzyme and Dipeptidyl Peptidase-IV Inhibitory Activities. J. Food Biochem..

[52] Chen J.-C., Wang J., Zheng B.-D., Pang J., Chen L.-J., Lin H.-T., Guo X. (2016). Simultaneous Determination of 8 Small Anti-hypertensive Peptides with Tyrosine at the C-Terminal in Laminaria japonica Hydrolysates by RP-HPLC Method. J. Food Process. Preserv..

[53] Annane D., Ouanes-Besbes L., de Backer D., Du B., Gordon A.C., Hernández G., Olsen K.M., Osborn T.M., Peake S., Russell J.A. (2018). A global perspective on vasoactive agents in shock. Intensive Care Med..

[54] Hayes M., Mora L., Lucakova S. (2022). Identification of Bioactive Peptides from Nannochloropsis oculata Using a Combination of Enzymatic Treatment, in Silico Analysis and Chemical Synthesis. Biomolecules.

[55] Måge I., Böcker U., Wubshet S., Lindberg D., Afseth N. (2021). Fourier-transform infrared (FTIR) fingerprinting for quality assessment of protein hydrolysates. LWT.

